# Ab Initio Computational Study of Chromate Molecular Anion Adsorption on the Surfaces of Pristine and B- or N-Doped Carbon Nanotubes and Graphene

**DOI:** 10.1186/s11671-017-1846-x

**Published:** 2017-01-25

**Authors:** Yuriy Hizhnyi, Sergii Nedilko, Viktor Borysiuk, Andrii Shyichuk

**Affiliations:** 1 Faculty of Physics, Taras Shevchenko National University of Kyiv, 64/13 Volodymyrska St., 01601 Kyiv, Ukraine; 2 Department of Rare Earth, Faculty of Chemistry, Adam Mickiewicz University, Umultowska 89b, 61-614 Poznań, Poland; 3 Faculty of Chemistry, University of Wrocław, Joliot-Curie 14, 50-383 Wrocław, Poland

**Keywords:** Excited states, CNT, Graphene, Chromate, TD-DFT, Binding energy, Adsorption

## Abstract

Density functional theory (DFT) computations of the electronic structures of undoped, B- and N-doped CNT(3,3), CNT(5,5) carbon nanotubes, and graphene with adsorbed chromate anions CrO_4_^2−^ were performed within molecular cluster approach. Relaxed geometries, binding energies, charge differences of the adsorbed CrO_4_^2−^ anions, and electronic wave function contour plots were calculated using B3LYP hybrid exchange-correlation functional. Oscillator strengths of electronic transitions of CrO_4_^2−^ anions adsorbed on the surfaces of studied carbon nanostructures were calculated by the TD-DFT method. Calculations reveal covalent bonding between the anion and the adsorbents in all studied adsorption configurations. For all studied types of adsorbent structures, doping with N strengthens chemical bonding with CrO_4_^2−^ anions, providing a ~2-eV increase in binding energies comparatively to adsorption of the anion on undoped adsorbents. Additional electronic transitions of CrO_4_^2−^ anions appear in the orange-green spectral region when the anions are adsorbed on the N-doped low-diameter carbon nanotubes CNT(3,3) and CNT(5,5).

## Background

Industrial-scale removal of heavy metals from environment is an urgent technological requirement of human civilization. One of the most promising methods of such kind of removal is based on adsorption of heavy metals on artificial adsorbents [1, 2]. The search for new adsorbent materials which can allow economically-efficient schemes of the heavy metal removal is a topical research task [1, 3]. Materials based on carbon nanostructured materials, in particular carbon nanotubes (CNTs) are considered as very promising adsorbents for such application [1, 2, 4, 5]. Among other adsorbent materials, the CNTs are characterized by a number of advantages, most notably large surface area and generally high adsorption capability of carbon surface for many toxic molecules [1, 3].

Removal of molecules which comprise Cr(VI) anions is of particular importance since these compounds are widely-spread industrial pollutants and, at the same time, are very harmful for living organisms [6, 7]. The properties of CNTs as adsorbent materials of Cr(VI) compounds were intensively studied within the last decade [4, 6, 8–12]. The CNT-based materials have been generally recognized to remove Cr(VI) efficiently [2, 6, 9–11]. However, at the single-molecule level, there is a considerable lack of understanding of Cr(VI)-on-CNTs adsorption mechanisms in spite of a large volume of accumulated experimental data. In particular, the peculiarities of chemical bonding between the most common types of Cr(VI) molecules and the CNT surface still remain unclear. Adsorption models developed in the mentioned above research papers usually rely on analogies with adsorption of molecules on a carbon surface without explicit consideration of specific CNT structures. However, it is well known that carbon surface of CNTs (particularly, low-diameter ones) is significantly curved and this feature determines their distinctive adsorption properties [13]. The mentioned lack of knowledge can be successfully remedied by the first-principles electronic structure calculations.

In recent years, such calculations have become a powerful tool for quantitative description of adsorption characteristics of various molecules on carbon materials, in particular CNTs and graphene sheets [14]. Adsorption of many kinds of molecules on CNTs of various structures had been considered so far in such computational studies (see [14] and references therein), and high predictive power of this theoretical method is now generally accepted. However, to the best of our knowledge, adsorption of Cr(VI) molecules on carbon nanostructured compounds (CNTs, fullerenes, graphene) has never been studied so far by the electronic structure calculations. This paper presents the first attempt of such study.

Here, we present results of the electronic structure calculations and related analysis for adsorption of CrO_4_^2−^oxyanions on surfaces of several types of carbon nanostructures. Geometry-optimized calculations were carried out at the density functional theory (DFT) level. Along with Cr_2_O_7_^2−^ and HCrO_4_^−^, CrO_4_^2−^ anions are a wide-spread kind of Cr(VI) compounds and can exist in aqueous solutions in a wide range of concentrations [6, 10]. Three kinds of CNT adsorbents were considered in the calculations: CNT(3,3), CNT(5,5), and graphene sheets. The two former materials were studied to clarify the adsorption mechanisms on low-diameter CNTs, while graphene sheets were considered as a model approximation for large-diameter CNTs. Together with undoped carbon materials, the B- or N-doped CNTs and graphene were considered in calculations. Such kind of doping can substantially change the physical properties of carbon nanostructures (see e.g., [15–17]). As it was found for several kinds of molecules, the doping with nonisovalent impurities B or N can enhance adsorption capabilities of carbon nanostructures [18–20]. This was the reason to examine the influence of B/N-doping on CNT adsorption capability with respect to CrO_4_^2−^ anions.

The calculation results showed that, for all of the studied adsorption cases, CrO_4_^2−^ anions form stable chemical bonds with carbon atoms (as well as B or N dopant atoms) of the CNT (or graphene) surface with binding energies ranging from about −2 to about −8 eV. The calculations predict that both pristine and B- or N-doped CNTs can act as efficient adsorbent materials for removal of CrO_4_^2−^ anions from gaseous phase (from air).

The paper also presents computational results on the energies of the electronic excited states (and corresponding oscillator strengths of transitions between the ground and the excited states) of CrO_4_^2−^ anions, in all of the studied adsorption cases. Optical absorption and luminescence of chromate anions are well studied and it was suggested that only significantly distorted CrO_4_^2−^ anions are able to emit light in visible spectral range [21–23]. The distortions split symmetry-degenerate excited electronic levels thus increasing the number of symmetry-allowed electronic transitions relatively to “free” CrO_4_^2−^ anion which has *T*_*d*_ point symmetry. Our calculations showed that, as CrO_4_^2−^anions are adsorbed on the N-doped low-diameter CNTs, they are significantly distorted. Moreover, additional optical absorption bands appear in the orange-green spectral. The calculations thus predict that both absorption and luminescence spectroscopy can be a useful tool for monitoring of CrO_4_^2−^ adsorption by N-doped CNT-based materials.

## Methods

**Fig. 1:**
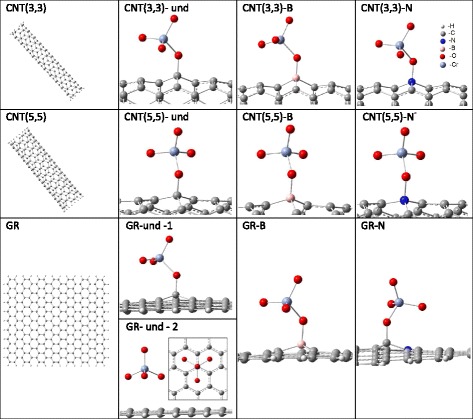
Geometry-optimized structures: undoped clusters (*left column*) and clusters with adsorbed CrO_4_^2−^ anions (other plots, only central parts of the clusters are shown)

The CNTs and graphene sheets were modeled as molecular clusters. The clusters of CNT(3,3) contained 126 carbon and 12 hydrogen atoms (see Fig. 1). They comprised 23 C_6_ “rings” of armchair carbon nanotube (3,3), while the dangling C-C bonds were capped with H atoms. Such a molecule is a widely used approximation in computational modeling of CNTs within molecular cluster approach (see e.g., [24]). Clusters with 21 C_10_ and 2 H_10_ rings were constructed to model CNT(5,5) providing a C_210_H_20_ formula. The C_266_H_46_ clusters were used to model graphene (GR) sheets (see corresponding plots in the left column of Fig. 1). In order to model the B_C_ or N_C_ substitutional impurities, one C atom from the central region of the clusters was substituted with either B or N.

The geometry optimization calculations were performed in vacuo using Gaussian 09 (build E01) software package and B3LYP nonlocal exchange-correlation density functional [25–27]. The split-valence double-zeta (6-31G) basis sets [28] were used for C and H atoms, while correlation-consistent polarized valence double-zeta (cc-pVDZ) basis sets [29] were applied for Cr and O atoms. Such combination of basis sets is generally utilized in computational studies of adsorption of molecules with d-metals on the CNT surfaces (see e.g., [30, 31]). All other settings were Gaussian 09 defaults.

Clusters of undoped CNT(3,3), CNT(5,5), and GR were considered in calculations as electrically neutral, while additional charges equal to −1*e* and 1*e* were assigned to clusters describing the B- and N-doped clusters, respectively. By using such additional charges, we have modeled a very common situation when an “additional” electron introduced into a real CNT by the N_C_ impurity is compensated by an additional charge +1*e* of defect created somewhere far from the N_C_ site at the stage of synthesis (such compensating defects were not modeled explicitly in our clusters). Correspondingly, additional compensating defects can supply one “missing” electron in the B_C_ case. Taking into account the −2*e* charge of free CrO_4_^2−^ anion, additional −2e charges were assigned to the clusters of undoped adsorbent and chromate anion. Consequently, additional charges of −3*e* or −1*e* were assigned to the B- or N-doped clusters, respectively.

In the geometry optimization studies, several starting positions of CrO_4_^2−^ anions with respect to the carbon surface were considered for each adsorption case. In all cases, the anions were initially put in such a way that the shortest C/B/N − O distance lied between 1.5 and 2.2 Å. Using such initial geometry, we ensure the binding of the anion particularly to the dopant atoms, not at just some other site of the doped CNT or graphene.

The following characteristic size parameters were obtained in geometry-optimized calculations for undoped CNT(3,3) cluster: distance along the nanotube axis between the outermost C_6_ rings was ~24.7 Å, distance between outermost H_6_ rings (cluster length) was ~26.5 Å, mean distance between “contralateral” C nuclei of the central C_6_ ring (cluster diameter) was ~4.21 Å. Analogously, the length and diameter of undoped CNT(5,5) cluster were ~26.5 and ~6.84 Å, respectively. Characteristic dimension of undoped GR cluster (the maximal internuclear distances between the H nuclei located on the opposite sides of the sheet) was ~24.1 × 30.8 Å.

It should be noted that the lengths of real CNTs usually exceed their diameters by several orders of magnitude. So, a “section” of the CNT modeled in a cluster must be long enough to guarantee a negligible influence of side surfaces on the central region of the section where the B(N) impurities and adsorbed CrO_4_^2−^ anions are situated. To examine this property, we have performed supplementary geometry optimization calculations of undoped CNT(3,3), CNT(5,5), and GR clusters. As the calculations showed, removal of the two outermost carbon rings from both edges of CNT(3,3) and CNT(5,5) clusters (or the two outermost carbon “strips” from GR cluster) changes the C-C distances in the central regions of clusters within 0.3%. Such slight changes ensure a negligible influence of the cluster edges on the calculated adsorption geometries. Consequently, the selected sizes of clusters were considered optimal, as larger clusters require more CPU time.

The binding energies *E*_*b*_ of chromate anions to adsorbents were obtained as the difference of the calculated total energies using the expression:

$$ {E}_b={E}_{\mathrm{CNT}-\mathrm{B}\left(\mathrm{N}\right)-{\mathrm{CrO}}_4^{2-}}-{E}_{\mathrm{CNT}\hbox{-} \mathrm{B}\left(\mathrm{N}\right)}-{E}_{{\mathrm{CrO}}_4^{2-}} $$

where ${E}_{\mathrm{CNT}-\mathrm{B}\left(\mathrm{N}\right)-{\mathrm{CrO}}_4^{2-}}$ is total energies of the optimized adsorption system “adsorbent with ${\mathrm{CrO}}_4^{2-}$ anion”, *E*_CNT − B(N)_is total energies of the optimized adsorbent, and ${E}_{{\mathrm{CrO}}_4^{2-}}$ is total energies of optimized ${\mathrm{CrO}}_4^{2-}$ anions calculated within the same approximations.

The calculated *E*_*b*_ value should be negative in a stable adsorption configuration. Differences in charge density on the CrO_4_^2−^ anion (in respect to the isolated anion) in adsorbed state were defined as ∆*q* = −2*e* − *q*, where *q* is an algebraic sum of charge states of Cr and O atoms (calculated in adsorbed configuration using the Mulliken population analysis, $q={q}_{{\mathrm{O}}_1}+{q}_{{\mathrm{O}}_2}+{q}_{{\mathrm{O}}_3}+{q}_{{\mathrm{O}}_4}+{q}_{\mathrm{Cr}}$). If the calculated value of ∆*q* is negative, the electronic charge is transferred from the anion to the adsorbent.

Excited electronic states of the free CrO_4_^2−^ anion (*T*_*d*_ point symmetry, ${R}_{\mathrm{Cr}-\mathrm{O}}^{\mathrm{free}}=1.65511\mathring{A}$) and in the adsorbed configurations were studied by the time-dependent DFT method (TD-DFT). In these calculations, the two-level ONIOM-2 approach was used [32]. The “adsorbent + CrO_4_^2−^ anion” system with optimized geometry was divided into two regions, the quantum mechanical (QM) and the molecular mechanical (MM). The QM region comprised the atoms of CrO_4_^2−^ anion, while the MM region comprised all atoms of the adsorbent molecule (C and H). The electronic embedding was used in order to take into account electrostatic interaction between the QM and MM regions, i.e., the atoms of the QM region were treated by TD-DFT calculations, while the atoms of the MM region were treated as partial charges contributing to the quantum-mechanical Hamiltonian. Successful applications of the ONIOM approach to calculations of the excited states of molecules are reviewed in [33]. The TD-DFT is a well-established method for treatment of the excited states of tetrahedral oxyanions of d^0^ metals (see [34] and references therein).

The TD-DFT calculations of the singlet excited states of CrO_4_^2−^ anions (QM region) were carried out in single-point calculation with the previously optimized geometry. The B3LYP exchange-correlation functional was used and only single excitations were taken into account. The same basis sets as for the geometry optimization calculations were used. Energies and oscillator strengths of electronic transitions from the ground to 50 lowest energy excited states were calculated. Additional details on computational procedures and corresponding references can be found on the official Gaussian 09 website [35].

## Results and Discussion

Calculated binding energies, charge differences of the adsorbed chromate anions, and the optimized shortest internuclear distances for studied adsorption configurations are presented in Table 1. Corresponding optimized structures are presented in Fig. 1. Two stable configurations were found for adsorption on the undoped graphene (denoted as GR-und-1 and GR-und-2), while each of the other adsorption cases revealed a single stable configuration. As the table shows, the binding energies for all studied adsorption cases range from about −1.8 to about −7.8 eV (per cluster). Such values of *E*_*b*_ are typical for the chemisorption mechanism (see e.g., [36]). In the GR-und-2 case, one of the faces of the CrO_4_^2−^ tetrahedron is parallel to the graphene surface. In all of the other cases, the tetrahedron is tilted, one of the O atoms of the chromate anion is located closer to the adsorbent surface and forms a single bond with it. As it is clearly illustrated by Fig. 1, the bonds are formed between one O atom of the anion and C, B, or N atom of the adsorbent.

**Table 1:** Calculated binding energies *E*_*b*_ (eV), charge differences of adsorbed CrO_4_^2−^ anions Δ*q* (e), and internuclear distances (Å)

Type of doping	Configuration		*E*_*b*_	∆*q*	(*R*_C-O_)^min^	(*R*_B(N)-O_)^min^	*R* ^1^_Cr-O_	(*R*_C-Cr_)^min^	(*R*_B(N)-Cr_)^min^
Undoped	CNT (3,3)-und		−4.84	−0.83	1.4161	–	1.8377	2.9312	–
	CNT (5,5)-und		−4.71	−0.9	1.4365	–	1.8538	2.9093	–
	GR-und	1	−3.93	−0.97	1.4678	–	1.8507	3.0149	–
		2	−3.57	−0.82	3.1343 3.1465 3.2281	–	1.6566 1.6576 1.6421	3.5174	–
B-doped	CNT (3,3)-B		−1.77	−0.59	2.5342	1.4481	1.7887	3.5543	2.9702
	CNT (5,5)-B		−2.3	−0.64	2.5608	1.4759	1.8014	3.4881	2.925
	GR-B		−2.54	−0.81	2.4693	1.5179	1.8067	3.3706	3.0236
N-doped	CNT (3,3)-N		−7.37	−1.11	2.3351	1.4807	1.8844	3.5806	2.9706
	CNT (5,5)-N		−6.68	−1.12	2.441	1.5197	1.8917	3.3126	2.9419
	GR-N		−7.85	−0.98	1.4075	2.3792	1.861	2.9778	3.3815

*(R*_*C-O*_ *)* ^*min*^, *(R*_*C-Cr*_ *)* ^*min*^, *(R*_*B(N)-O*_ *)* ^*min*^, *(R*_*B(N)-Cr*_ *)* ^*min*^ shortest internuclear distances between atoms of adsorbents (C, B, or N) and atoms of CrO_4_^2−^ anions (O or Cr), *R* ^*1*^_*Cr−O*_ internuclear distances between closest to the adsorbent O atoms and Cr atoms

**Fig. 2:**
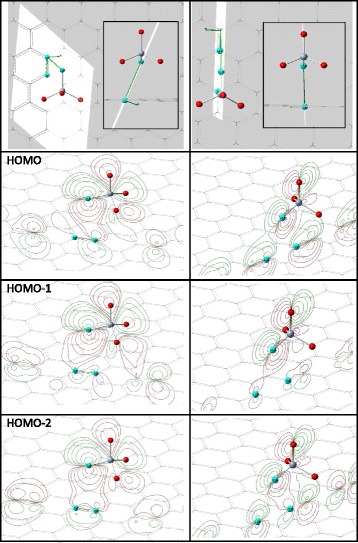
Contour plots of selected molecular orbitals for GR-und-2 adsorption configuration calculated in two planes (*left and right columns*); the highest occupied molecular orbital (HOMO) and the two MOs under it; quasi-orthogonal images of the planes are given in the upper row; in-plane nuclei are highlighted in *blue*

A more complex bonding picture is observed for GR-und-2 configuration. In this case, the ${R}_{\mathrm{C}-\mathrm{O}}^{\min }$ distance is much longer (~3.13 Å, see Table 1) and as many as three O atoms are bonded to C atoms of the adsorbent (internuclear distances for two additional O atoms are also listed in corresponding cell of Table 1). The bonding peculiarities for one of these three O atoms are illustrated by the electronic wavefunction contour plots (see Fig. 2).

As Fig. 1 shows, for GR-und-2 configuration, each of the three O atoms is located about 2.80 Å above the center of C_6_ “honeycomb” of graphene surface (see the inset in the corresponding plot) and all three O atoms have approximately the same local surrounding (within 0.01 Å accuracy of nuclear coordinates). Within this accuracy, the structure is characterized by the C_3_ symmetry axis perpendicular to the graphene plane and containing one C atom of the adsorbent, the Cr atom, and the “upper” O atom of the CrO_4_^2−^ anion (see the inset in Fig. 1). The planes in Fig. 2 were selected to illustrate the bonding between one O atom and all six C atoms of the honeycomb. The plane in the left column of Fig. 2 contains one O atom of the anion and two C atoms of the honeycomb. The right column of Fig. 2 presents a plane containing the same O atom and two other C atoms of the honeycomb. The bonding between the O atom and the two remaining C atoms of the honeycomb is similar to the left plane, so corresponding pictures are not presented here. The left column of Fig. 2 clearly indicates overlap between *p*-orbitals of the O and one of the C atoms (HOMO and HOMO-1 cases) as well as between the O and simultaneously two C atoms (HOMO-2 case). The right column of Fig. 2 indicates overlap between *p*-orbitals of the O atom and two other C atoms of the honeycomb. Thus, for GR-und-2 configuration, each of the three O atoms of the anion interacts with six C atoms of graphene. A substantial redistribution of the electronic wavefunctions of the CrO_4_^2−^ anion and the adsorbent indicates substantial strength of this interaction.

**Fig. 3:**
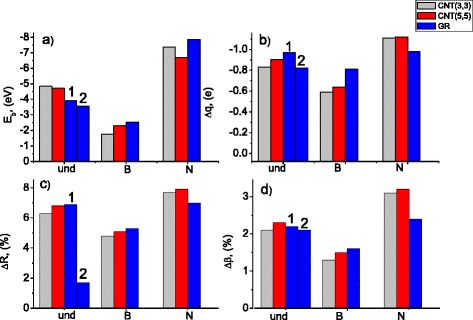
Dependencies of the binding energies (**a**), charge differences of adsorbed CrO_4_^2−^ anions (**b**), and characteristic parameters of CrO_4_^2−^ distortions (**c, d**) on type of the adsorbent (indicated by *column colors*) and type of the adsorbent doping (see notations below column groups)

For convenience of analysis of the *E*_*b*_ and Δ*q* dependence on the type of adsorbent (CNT(3,3), CNT(3,3), or GR) and the type of doping (N or B), the corresponding data from Table 1 are presented as column diagrams in Fig. 3. As Fig. 3 shows, doping of the studied adsorbents with boron reduces the absolute value of the binding energy almost twice (averagely, by ~2 eV), significantly weakening the chemical bonding between the anions and the adsorbents. Doping with nitrogen, in turn, strengthens the bonding: corresponding *E*_*b*_ values are significantly higher (averagely, by ~2 eV). The Δ*q* columns (Fig. 3b) reveal the same tendencies as for *E*_*b*_ values. The decrease in charge transferred to the adsorbent (averagely, by ~0.2*e*) is observed in case of B-doping, whereas for N-doped adsorbents, this charge increases by ~0.2*e* comparing to the cases of undoped adsorbents.

Such significant changes of the binding energy lead to the following assumption: both B- and N-doping could be an efficient tool for tuning of the adsorption properties of carbon nanostructures with respect to the CrO_4_^2−^ anions.

As Fig. 3a shows, adsorption of CrO_4_^2−^ anions on undoped low-diameter CNTs (3,3) and (5,5) is characterized by higher absolute values of binding energies than adsorption on undoped graphene sheets. However, for the B- and N-doped cases, correlations between the type of adsorbent and calculated *E*_*b*_ value are less evident. Dependencies of *E*_*b*_ on adsorbent type do not correlate with corresponding dependencies of Δ*q* for undoped and N-doped adsorbents (compare the differences in column heights within corresponding groups of the columns in Fig 3a, b). One can conclude that the adsorbent structure (in particular, tube diameter, since graphene sheets can be considered as an approximation for high-diameter CNTs) is a less important factor for tuning of the adsorption properties of carbon nanostructures with respect to CrO_4_^2−^ anions if compared with B(N)-doping influence. In other words, in order to create an efficient carbon-based adsorbent for chromate anions, chemical modification of the material is crucial, while the size of CNTs is not that much important. Another conclusion is that no separation of CNTs by diameter or length is required; a mixture of CNTs and graphene fragments might be a good adsorbent, given an appropriate chemical tuning. However, additional studies of a wider set of CNT structures and anions would be necessary to prove these assumptions.

In all of the presented cases, adsorbed chromate anion transfers a substantial value of electronic charge (ranging from about −0.6 to about −1.1*e*, see Table 1) to the adsorbents. Such valuable charge is usually obtained for the chemisorption mechanism of adsorption (see e.g., [36]).

The data on geometrical distortions of adsorbed CrO_4_^2−^anions comparatively to free chromate anions are presented in Fig. 3c, d. Characteristic parameters of distortions of adsorbed CrO_4_^2−^ anions were calculated as the following:

$$ \varDelta R\left(\%\right)=\frac{\sqrt{\frac{1}{4}{\displaystyle {\sum}_{i=1}^4}{\left({R}_{\mathrm{Cr}-\mathrm{O}}^i-{R}_{\mathrm{Cr}-\mathrm{O}}^{\mathrm{free}}\right)}^2}}{R_{\mathrm{Cr}-\mathrm{O}}^{\mathrm{free}}}100\% - \mathrm{r}\mathrm{elative}\ \mathrm{standard}\ \mathrm{deviation}\ \mathrm{of}\ \mathrm{C}\mathrm{r}\ \hbox{--}\ \mathrm{O}\ \mathrm{distances}\ {R}_{\mathrm{Cr}-\mathrm{O}}; $$

$$ \varDelta \beta \left(\%\right)=\frac{\sqrt{\frac{1}{4}{\displaystyle {\sum}_{i=1}^4}{\left({\beta}^i-{\beta}^{\mathrm{free}}\right)}^2}}{\beta^{\mathrm{free}}}100\% - \mathrm{r}\mathrm{elative}\ \mathrm{standard}\ \mathrm{deviation}\ \mathrm{of}\ \mathrm{O}\ \hbox{--}\ \mathrm{C}\mathrm{r}\ \hbox{--}\ \mathrm{O}\ \mathrm{angles}\ \upbeta; $$

As Fig. 3c, d shows, the degree of distortions (for both distances and angles) distinctly depends on the type of doping and has no evident correlation with the adsorbent structure. Only the GR-und-2 configuration does not conform to the tendency since it is characterized by far different adsorption geometry (see Fig. 2 and accompanying text). When the anions are adsorbed on the B-doped adsorbents, they undergo weaker distortions as compared to adsorption on undoped structures. In turn, the distortions become stronger when the anions are adsorbed on the N-doped carbon nanostructures. The picture of distortions is well consistent with the indicated above decrease (increase) of the strength of chemical bonding due to B(N)-doping.

So as we showed above, distortion and charge transfer from to the adsorbent are the most noticeable manifestations of the carbon nanostructure effect on the CrO_4_^2−^ anion. It is obvious that, due to adsorption on a carbon surface, the spectrum of electronic transitions of CrO_4_^2−^ anion can also be significantly changed as compared to corresponding spectrum of the anion in vacuum. Actually, geometry distortions and the charge transfer are inseparable manifestations of the same physical process of adsorption. Here, we have artificially separated the influence of these two factors on the structure of excited states and electronic transition probabilities. In many cases, considering only geometrical distortions was a quite productive approximation in computational studies of the electronic structure of CrO_4_^2−^ anions, as well as in interpretation of experimental results [21–23]. In particular, geometrical distortions were considered as factor that determines characteristics of the CrO_4_^2−^ anion luminescence in the visible spectral region. As a result of the studies of CrO_4_^2−^ anions located in chromate or sulfate crystals or deposited on the surface of dispersed SiO_2_, it was concluded that the luminescence of these materials in the visible spectral region originates from only those CrO_4_^2−^ which are located near structural defects, i.e., from those anions that underwent significant geometrical distortions [22, 23]. Lowering of the local symmetry of the anions can lift the symmetry restriction of radiative electronic transitions and therefore can lead to increase in luminescence intensity.

Effect of surrounding molecules in condensed media on the lowest energy excited states of tetrahedral oxyanions of d^0^ metals is currently a matter of dispute (in particular, if this effect is modeled by electrostatic influence of solvent, see [34] and references therein). In particular, it was argued that the influence of surrounding solvent on the structure of excited states of the tetrahedral oxyanions is rather weak since optical absorption spectra of the oxyanions in liquids are in general similar to their spectra in gas phase [37, 38].

Below, we consider two cases. (A) The distortions of chromate anions are only taken into account. The excited states of the isolated anions are calculated in vacuum, while their geometries are those corresponding to the adsorbed anions, as modeled above. (B) The influence of carbon adsorbents was taken into account in calculations of the excited states using the ONIOM model, as described in “Methods” Section. Comparing the results for these two cases, we can estimate the strength of influence of particular adsorbents on the CrO_4_^2−^ excited state structure. In our opinion, this issue may have some importance to the problem of computational description of the excited states of d^0^ metal oxyanions.

**Fig. 4:**
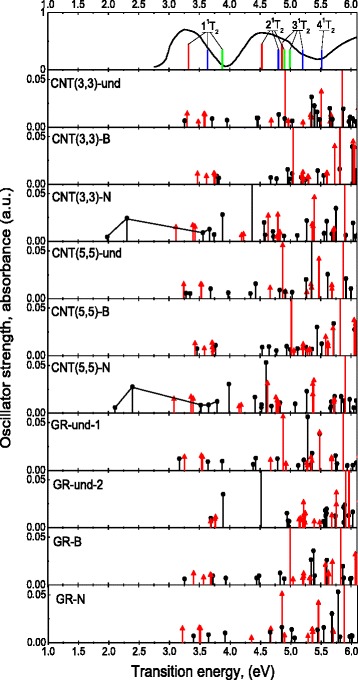
Highest plot: calculated energies of electronic transitions from the ground (^1^A_1_) to the low-energy excited states (the assignment is indicated in the plot) of free CrO_4_^2−^ anions calculated by us (*blue bars*); energies of the same transitions calculated with another basis sets [34] (*green bars*) and determined experimentally (*red triangles*); experimental absorption spectrum of chromate anion in aqueous solution (*black solid line*) [39]. Other plots: oscillator strengths of electronic transitions of CrO_4_^2−^ anions adsorbed on the surface of carbon nanostructures calculated with (*black circles*) and without (*red triangles*) account of influence from the adsorbents

Changes in the spectra of electronic transitions in the CrO_4_^2−^ anions due to their adsorption on the surface of carbon adsorbents are presented in Fig. 4. For convenience of analysis, only transitions with oscillator strengths greater than 0.005 are given. For comparison, we also present the excitation energies of free CrO_4_^2−^ anion calculated by the TD-DFT/B3LYP/TZ2P method [34] (the same method and correlation functional as in our calculation, but another basis set was used), as well as the energies of electronic transitions of CrO_4_^2−^ anion determined from experimental spectra as peak positions of the optical absorption bands [39]. These experimental energy values are frequently used for comparison with calculation results (see e.g., [34]).

As Fig. 4 (uppermost plot) shows, our calculation results for energies of ^1^A_1_ → 1^1^T_2_, ^1^A_1_ → 2^1^T_2_, ^1^A_1_ → 3^1^T_2_, and ^1^A_1_ → 4^1^T_2_ transitions for free CrO_4_^2−^ anion overestimate corresponding experimental energies approximately by the same value of ~0.3 eV for each of them. At the same time, e.g., the ^1^A_1_ → 2^1^T_2_ energy in our calculation is much closer to the experimental value than it was obtained in [34]. Despite observed differences, we can argue that our results quite well reproduce the experimental transition energies of free CrO_4_^2−^. Observed discrepancies are of order that was commonly observed in recent calculations of the excited states of tetrahedral oxyanions of d-metals [34, 40].

The spectra of adsorbed chromate anions (see lower plots in Fig. 4) present more allowed transitions than it was observed for free CrO_4_^2−^, even if the calculations took into account only geometry distortions of the anions (see red triangles in Fig. 4). The number of nonzero oscillator strengths in the spectra increases since the distortions lift the symmetry restrictions and substantially split all of the mentioned above ^1^T_2_ degenerate states of the free anion. The low-energy parts of the spectra for each adsorption case contain a group of at least three transitions corresponding to the lowest energy transition ^1^A_1_ → 1^1^T_2_ of free anion. At that, the lowest energy transition for the cases of undoped adsorbents (3.30, 3.24, and 3.25 eV for CNT(3,3), CNT(5,5), and GR-und-1 cases, correspondingly), except GR-und-2, lie closer to the experimental value (3.32 eV) than the calculated lowest energy transition ^1^A_1_ → 1^1^T_2_ of free anion (see Fig. 4).

The positions of the lowest energy transitions correlate with the degree of the anion distortions. Regardless of the adsorbent structure, the lowest energy transition is shifted by several tenths of electronvolt to higher energies for the B-doped adsorbents, whereas it is shifted by several tenths of electronvolt to lower energies for the N-doped structures (the GR-und-2 case is an exception due to described above peculiarities of bonding).

When the electrostatic influence from the adsorbents is taken into account (see black circles in Fig. 4), the changes in the oscillator strength spectra become more valuable. However, only for adsorption on the nitrogen-doped CNT(3,3)-N and CNT(5,5)-N, additional allowed transitions appear far outside the experimental absorption spectrum of chromate anion. In both cases, the oxygen atom of the anion is bonded to the N atom of the adsorbent (see Table 1 and Fig. 1). These additional transitions are observed in the orange-green optical range, near 2.0 and 2.3 eV (see Fig. 4). Such additional transitions are not observed for the GR-N case for what the oxygen atom of the anion is bonded to the C atom rather than to the N atom of the adsorbent. As it follows from Table 1, these two cases are characterized by the highest (among all cases studied) values of electronic charge transferred from the anion to the adsorbent, namely, −1.11 and −1.12 for CNT(3,3)-N and CNT(5,5)-N, respectively. So, the effect of the charge transfer from CrO_4_^2−^ anion to the adsorbent is a very important factor that influences the spectral properties of the “CrO_4_^2−^-carbon nanostructure” system. This is not surprising since, as it was testified by various calculation methods, the energies of the lowest energy transitions in CrO_4_^2−^ anions imply charge-transfer from O 2*p* to Cr 3*d* orbitals and therefore these energies should be sensitive to changes in charge states of the oxygen atoms [34, 37].

Our computational results assume that spectroscopy might be useful to monitor CrO_4_^2−^ adsorption by the N-doped CNT-based materials, since in this case, the adsorbed anions could reveal additional absorption bands, which are red-shifted by ~1 eV comparing to corresponding spectral bands of free anions. However, further studies are obviously required to prove this assumption.

## Conclusions

Computational studies of adsorption of CrO_4_^2−^ anions on undoped, B- and N-doped carbon nanostructures CNT(3,3), CNT(5,5), and graphene reveal covalent bonding between the anion and the adsorbents for all studied adsorption cases. One O atom of the anion is bonded to either C, B, or N atom of the adsorbent in all cases, except for the GR-und-2, where three O atoms of the CrO_4_^2−^ anion are characterized by overlapped electronic wavefunctions with a total of 13 C atoms of the adsorbent. A valuable amount of electronic charge (ranging from ~0.6 to ~1.1*e*) is transferred from CrO_4_^2−^ anion to the adsorbent in all adsorption cases. For all studied types of adsorbent structures, doping with N strengthens chemical bonding with CrO_4_^2−^ anions: the binding energies increase averagely by ~2 eV in comparison with adsorption on corresponding undoped adsorbents. Adsorbtion of the chromate anion on the N-doped low-diameter carbon nanotubes CNT(3,3) and CNT(5,5) results in additional electronic transitions of CrO_4_^2−^ anions in the green spectral region of the absorption spectrum. At the same time, adsorption of the CrO_4_^2−^ anion on other adsorbents considered here has no such significant influence on its optical absorption spectrum.

## Abbreviations

cc-pVDZ: Correlation-consistent polarized valence double-zeta
CNTs: Carbon nanotubes
DFT: Density functional theory
GR: Graphene
HOMO: Highest occupied molecular orbital
MM: Molecular mechanical
ONIOM: Our own n-layered integrated molecular orbital and molecular mechanics
QM: Quantum mechanical
TD-DFT: Time-dependent density functional theory
